# High nuclear/cytoplasmic ratio of Cdk1 expression predicts poor prognosis in colorectal cancer patients

**DOI:** 10.1186/1471-2407-14-951

**Published:** 2014-12-15

**Authors:** Wen-Wei Sung, Yueh-Min Lin, Pei-Ru Wu, Hsu-Heng Yen, Hung-Wen Lai, Tzu-Cheng Su, Ren-Hung Huang, Chun-Kai Wen, Chia-Yu Chen, Chih-Jung Chen, Kun-Tu Yeh

**Affiliations:** School of Medicine, Chung Shan Medical University, Taichuang, Taiwan; Department of Surgical Pathology, Changhua Christian Hospital, Changhua, Taiwan; Department of Medical Technology, Jen-Teh Junior College of Medicine, Nursing and Management, Miaoli, Taiwan; Department of Gastroenterology, Changhua Christian Hospital, Changhua, Taiwan; School of Medicine, National Yang Ming University, Taipei, Taiwan

**Keywords:** Cyclin-dependent kinase 1, Prognosis, Colorectal cancer

## Abstract

**Background:**

Cdk1 (cyclin-dependent kinase 1) is critical regulator of the G2-M checkpoint. Cyclin-dependent kinase pathways are considered possible targets for cancer treatment; however, the prognostic role of Cdk1 in colorectal cancer is still controversial. Therefore, we attempted to determine the impact of Cdk1 on the clinical outcome of colorectal cancer patients to further identify its role in colorectal cancer.

**Methods:**

Cdk1 immunoreactivity was analyzed by immunohistochemistry (IHC) in 164 cancer specimens from primary colorectal cancer patients. The medium follow-up time after surgery was 3.7 years (range: 0.01 to 13.10 years). The prognostic value of Cdk1 on overall survival was determined by Kaplan-Meier analysis and Cox proportional hazard models.

**Results:**

All samples displayed detectable Cdk1 expression with predominant location in the cytoplasm and nucleus. A high Cdk1 nuclear/cytoplasmic (N/C) expression ratio was correlated with poor overall survival (5-year survival rate: 26.3% vs 46.9%, N/C ratio ≥1.5 vs N/C ratio <1.5, log-rank p = 0.027). Accordingly, a Cdk1 N/C expression ratio ≥1.5 was identified as an independent risk factor by multivariate analysis (hazard ratio = 1.712, P = 0.039).

**Conclusions:**

We suggest that Cdk1 N/C expression ratio determined by IHC staining could be an independent prognostic marker for colorectal cancer.

**Electronic supplementary material:**

The online version of this article (doi:10.1186/1471-2407-14-951) contains supplementary material, which is available to authorized users.

## Background

Colorectal cancer is one of the major causes of cancer deaths in the world, and identifying reliable prognostic markers is still an important issue for improving patients' outcomes [1]. The clinical outcomes and treatment of patients with colorectal cancer mainly depend on tumor location and stage at diagnosis. Cancer metastasis, either before or after surgical resection, leads to significantly poor survival [2]. Successful metastasis requires that the cancer cells enter the blood circulation, attach to the endothelium, invade the target distant organs, and subsequently form tumors [3, 4]. Recently, we found that cyclin-dependent kinase 1 (Cdk1) was overexpressed at the RNA level in circulating tumor cells of colorectal cancer when compared with control cells. This prompted us to investigate the role of Cdk1 in the clinical outcome of colorectal cancer patients.

Molecules involved in cell cycle regulation have been attracting considerable attention as potential prognostic and therapeutic cancer markers [5, 6]. Cdks are key regulators of cell cycle and RNA transcription [6] and cell cycle defects in tumors are often mediated by altered Cdk activities that result in unscheduled proliferation and genomic and chromosomal instability [6]. Among Cdks, Cdk1 is sufficient for driving the cell cycle in all cell types, identifying its master role in regulating cell proliferation [6]. Cdk1, formerly called cell division control protein 2 (Cdc2), interacts with cyclin B1 to form an active heterodimer that drives cells through mitosis [7, 8].

Cdk1 has been identified as a clinically useful prognostic marker in non-small cell lung cancer, colon cancer, and breast cancer [9–15]. However, its prognostic role is still controversial and more evidence is needed for further clinical application. A high specific activity of Cdk1 measured on 254 fresh-frozen tumor samples from stage II colon cancer patients was associated with short distant-metastasis-free intervals and poor prognosis [14]. An association between high specific activity of Cdk1 and microsatellite-stable tumors provided molecular evidence to further support this finding [14]. However, Meyer and colleagues revealed that patients with an ‘absent’ score for Cdk1 had poor cancer-related 5-year survival, indicating the absence of Cdk1 to be an independent prognostic marker in stage UICC II colon carcinoma [15]. These conflicting results led us to investigate the role of Cdk1 in our patient population.

Interestingly, Zhang and colleagues found that Cdk1 was highly expressed in non-small cell lung cancer tumor tissues, but its loss from the cytoplasm could predict poor prognosis [13]. This finding prompted us to consider that the prognostic role of Cdk1 might depend on its expression in the nucleus and cytoplasm. The aim of the present study was to clarify the prognostic implications of Cdk1 expression in the nucleus and cytoplasm in colorectal cancer.

## Methods

### Ethics statement

In this study, we enrolled de-linked tissue specimens from 164 colorectal cancer patients and the written and oral consent was not obtained. Tissue collection was retrospective and all tissue was obtained from hospitals archives. The Institutional Review Board waived the need for consent. This study was approved by the Institutional Review Board and the Ethics Committee of the Changhua Christian Hospital, Changhua, Taiwan (IRB no. 121008).

### Study subjects

This study enrolled 164 colorectal cancer patients. The tumor tissues were collected from patients with confirmed histological diagnosis at Changhua Christian Hospital between 1997 and 2000. No patient underwent preoperative radiotherapy, chemotherapy, or any other treatment. Cancers were staged according to the AJCC Colon Cancer Staging, 7th edition (2009). Clinical data including sex, age, stage, T, N, and M stages, and follow-up information were obtained from medical records and the cancer registry.

### Immunohistochemistry staining and evaluation of Cdk1 immunoreactivity

Immunohistochemistry (IHC) staining was performed at the Department of Surgical Pathology, Changhua Christian Hospital, as previously described [16, 17]. IHC analyses were performed on tissue microarray sections (4 μm) of formalin-fixed, paraffin-embedded, pre-chemotherapy primary colorectal tumors. The sections were placed on coated slides, washed with xylene to remove the paraffin, and rehydrated through serial dilutions of alcohol, followed by washings with a solution of phosphate buffered saline, PBS (pH = 7.2). Endogenous peroxidase activity was blocked with 3% H_2_O_2_. Antigen retrieval was performed by boiling in citrate buffer (10 mM) for 20 min. After incubation with the anti-human Cdk1 antibody (Cdc2 p34 antibody, 1:180 dilution; sc-166135, Santa Cruz Biotechnology) for 20 min at room temperature and thorough washing (three times with PBS). The immunoreaction was visualized using polymer-based MACH4 DAB Detection Kit (Biocare Medical) according to the manufacturer’s instructions to obtain optimal immunoreactivity and least background artifact. The slides were incubated with a horseradish peroxidase (HRP)/Fab polymer conjugate for another 30 min. The sites of peroxidase activity were visualized using 3,3'-diamino-benzidine tetrahydrochloride as the substrate for 300 seconds and hematoxylin as the counterstain. Pathologically verified normal colon specimens were used as positive control (Additional file 1: Figure S1). PBS was used instead of primary antibodies as a negative control (Additional file 1: Figure S1). Immunoreactivity scores were analyzed by pathologists using scores defined as previously described [17, 18]. In brief, immunoreactivity scores were defined as the cell staining intensity (0 = nil; 1 = weak; 2 = moderate; and 3 = strong) multiplied by the percentage of stained cells (0–100%), leading to scores from 0 to 300. Cdk1 immunoexpression was assessed semiquantitatively by 2 pathologists (YML and CJC), who independently scored coded sections based on the staining score without knowledge of clinical and follow-up information. A final agreement was obtained for each score by using a multiheaded microscope (Olympus BX51 10 headed microscopes).

### Statistical analysis

The Student *t* test, Fisher's exact test and the χ^2^ test were applied for continuous or discrete data analysis. The associations between the Cdk1 and patient survival were estimated using the Kaplan–Meier method and assessed using the log-rank test. Potential confounders were adjusted by Cox regression models, with the Cdk1 fitted as indicator variables. Overall survival time was defined as the interval between the date of surgery and the date of last follow-up or death. All statistical analyses were conducted using the SPSS statistical software program (version 15.0) (SPSS, Inc., Chicago, IL). All statistical tests were 2-sided, and the values of *P* <0.050 were considered statistically significant.

## Results

### Cdk1 is expressed in the majority of colorectal specimen and locates to both the cytoplasm and nucleus

We verified the role of Cdk1 in clinical outcome of colorectal patients by recruiting 164 patients with primary tumors. The clinicopathological characteristics of the study subjects are listed in Table 1. The mean age was 64.5 ± 12.9 years (mean ± SD) and the gender ratio was 0.72: 1.00 (female: male). In total, 22 patients had stage I tumors, 64 patients had stage II tumors, 50 patients had stage III tumors, and 28 patients had stage IV tumors. Twenty-seven patients had distant metastasis at diagnosis.

**Table 1:** **Relationships of cytoplasm and nucleus Cdk1 expression with clinical parameters in colorectal cancer patients**

Parameters	Case number	Cytoplasm Cdk1 expression		p value	Nucleus Cdk1 expression		p value
		≦200	>200		≦180	>180	
Age (year)		64.1 ± 12.4	65.2 ± 13.9	0.582	64.5 ± 13.3	64.6 ± 12.7	0.948
Gender							
Female	69	37 (53.6)	32 (46.4)	0.054	33 (47.8)	36 (52.2)	0.940
Male	95	65 (68.4)	30 (31.6)		46 (48.4)	49 (51.6)	
Stage							
I	22	11 (50.0)	11 (50.0)	0.623	10 (45.5)	12 (54.5)	0.241
II	64	42 (65.6)	22 (34.4)		33 (51.6)	31 (48.4)	
III	50	31 (62.0)	19 (38.0)		19 (38.0)	31 (62.0)	
IV	28	18 (64.3)	10 (35.7)		17 (60.7)	11 (39.3)	
T value							
1	5	3 (60.0)	2 (40.0)	0.033	4 (80.0)	1 (20.0)	0.180
2	20	9 (45.0)	11 (55.0)		6 (30.0)	14 (70.0)	
3	124	76 (61.3)	48 (38.7)		61 (49.2)	63 (50.8)	
4	15	14 (93.3)	1 (6.7)		8 (53.3)	7 (46.7)	
N value							
0	94	58 (61.7)	36 (38.3)	0.101	46 (48.9)	48 (51.1)	0.096
1	63	37 (58.7)	26 (41.3)		27 (42.9)	36 (57.1)	
2	7	7 (100.0)	0 (0.0)		6 (85.7)	1 (14.3)	
M value							
0	137	84 (61.3)	53 (38.7)	0.600	63 (46.0)	74 (54.0)	0.207
1	27	18 (66.7)	9 (33.3)		16 (59.3)	11 (40.7)	

**Figure 1:**
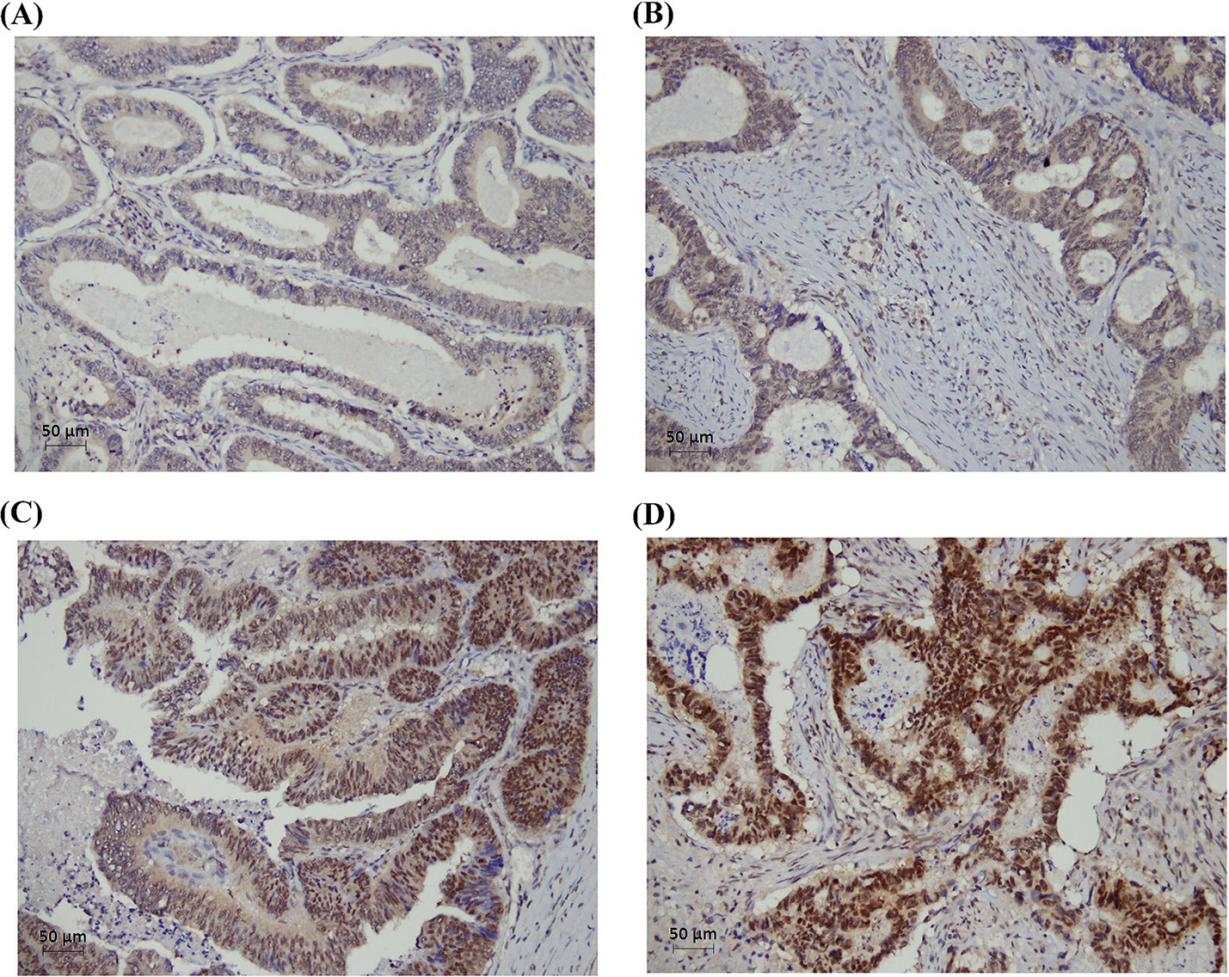
**Representative immunostaining of Cdk1 in colorectal cancer in tissue arrays according to the N/C ratio.** N/C ratio of Cdk1 were **(A)** 0.00-0.49; **(B)** 0.50-0.99; **(C)** 1.00-1.49; **(D)** ≥1.5. (Magnification: 200×).

Cdk1 expression was evaluated by IHC staining of tissue arrays. Figure 1 shows a representative immunostaining of Cdk1 in a colorectal cancer specimen. The Cdk1 expression in the cytoplasm and nucleus were scored separately by pathologists. All specimens had Cdk1 expression in cytoplasm and only 2 cases had no Cdk1 expression in the nucleus (1.2%). The median Cdk1 expression score was 200 for cytoplasm staining and 180 for nucleus staining and we used the median value as cut-off point for further analysis. As shown in Table 1, Cdk1 expression in the cytoplasm and nucleus had no significant association with age, gender, stage or TNM value, except that the patients with low cytoplasmic Cdk1 expression (score ≦200) were prone to have an advanced T value (p = 0.033). Overall, Cdk1 expression in the cytoplasm and nucleus was not related to clinical parameters except cytoplasm Cdk1 expression in T stage in our study population.

### The prognostic role of Cdk1 expression in the cytoplasm and nucleus of colorectal patients

We further verified the prognostic role of Cdk1 expression in colorectal patients. Overall survival data were collected and no data were missing among 164 patients. The mean and median follow-up times after surgery were 5.1 and 3.7 years (range from 0.01 to 13.10 years), respectively. The 5-year survival rate was 44.5%. During the survey, 138 patients died. We evaluated the possible correlation of Cdk1 expression and clinical outcome by grouping patients into four subgroups according to the quartile and median values of Cdk1 expression in the cytoplasm and nucleus (cut-off points were 180, 200, and 285 for the cytoplasm; and 150, 180, and 255 for the nucleus). Patients' survival was estimated using the Kaplan-Meier method and Cdk1 expression in cytoplasm and nucleus was not a significant prognostic marker in our study (log-rank test: p = 0.565 for cytoplasm expression and p = 0.688 for nucleus expression, Additional file 2: Figure S2). We further analyzed the prognostic value by Cox regression model, as shown in Additional file 3: Table S1. Neither cytoplasmic nor nuclear Cdk1 expression could predict clinical outcome in 5-year survival, univariate analysis, and multivariate analysis. Thus, we found no prognostic role of Cdk1 expression when examined separately in the cytoplasm and nucleus.

### High nuclear/cytoplasmic ratio of Cdk1 expression predicts poor prognosis in colorectal cancer

**Figure 2:**
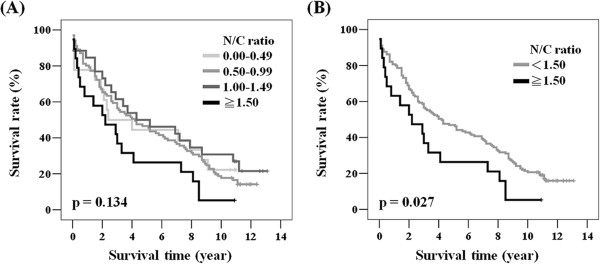
**A high Cdk1 N/C ratio was correlated with poor overall survival.** Kaplan-Meier actuarial analysis of overall survival according to **(A)** N/C ratio subgroups and **(B)** N/C ratio cut by 1.5 of Cdk1 immunostaining expression in colorectal cancer patients. The case number was 18, 101, 26, and 19 according to N/C ratio subgroups, respectively.

We considered the protein dynamics of posttranslational regulation by combining the cytoplasmic and nuclear expression of Cdk1 as a N/C ratio for further analysis [19]. The N/C ratio ranged from 0.0 to 24.0 (mean ± SD: 1.3 ± 2.2; medium: 0.9). The cut-off point (N/C ratio: 1.5) was determined according to the clinical outcome observed in different subgroups (Figure 2A), as shown in Figure 2B. Table 2 shows the relationships of the N/C ratio with clinical parameters. Patients with tumors with N/C ratios ≥1.5 had a significantly advanced T value compared with those with N/C ratios <1.5 (p = 0.004, Table 2). However, no significant difference was noted between patients with high and low N/C ratios with respect to tumor stage, N value, or M value.

**Table 2:** **Relationships of nucleus/cytoplasm ratio (N/C ratio) of Cdk1 expression with clinical parameters in colorectal cancer patients**

Parameters	Case number	Cdk1 N/C ratio		p value
		<1.5	≥1.5	
Age (year)		65.3 ± 12.6	58.8 ± 14.5	0.078
Gender				
Female	69	64 (92.8)	5 (7.2)	0.139
Male	95	81 (85.3)	14 (14.7)	
Stage				
I	22	20 (90.9)	2 (9.1)	0.975
II	64	56 (87.5)	8 (12.5)	
III	50	44 (88.0)	6 (12.0)	
IV	28	25 (89.3)	3 (10.7)	
T value				
1	5	5 (100.0)	0 (0.0)	0.004
2	20	18 (90.0)	2 (10.0)	
3	124	113 (91.1)	11 (8.9)	
4	15	9 (60.0)	6 (40.0)	
N value				
0	94	82 (87.2)	12 (12.8)	0.803
1	63	57 (90.5)	6 (9.5)	
2	7	6 (85.7)	1 (14.3)	
M value				
0	134	118 (88.1)	16 (11.9)	0.764
1	30	27 (90.0)	3 (10.0)	

We further analyzed the prognostic impact of the N/C ratio in our population. We grouped the N/C ratios into 4 subgroups consisting of the following values: 0.00-0.49; 0.50-0.99; 1.00-1.49; ≥1.50. Figure 2A shows patient survival estimated using the Kaplan-Meier method; patients with N/C ratios ≥1.5 had the poorest survival of all subgroups (p = 0.134). We estimated the prognostic significance of this N/C ratio, we compared the clinical outcome of patients with N/C ratios ≥1.5 and <1.5. Patients with N/C ratios ≥1.5 had significantly poorer survival compared with those with N/C ratios <1.5 (Figure 2B). The 5-year survival of patients with N/C ratios ≥1.5 was 26.3% while it was 46.9% in those with N/C ratios <1.5 (Table 3). We further confirmed our finding by Cox regression model (Tables 3 and 4). We adjusted age, gender, stage, and N/C ratio in multivariate analysis (Table 4). In our study population, patients with advanced stage cancer had significantly poorer prognosis compared with those with early stage disease (HR = 1.783, 95% CI = 1.071-2.966, P = 0.026 for univariate analysis; HR = 1.712, 95% CI = 1.027-2.853, P = 0.039 for multivariate analysis, Tables 3 and 4). As expected, significantly poorer clinical outcome was found for patients with N/C ratios ≥1.5 (HR = 1.733, 95% CI = 1.051-2.856, P = 0.031 for univariate analysis; HR = 1.712, 95% CI = 1.027-2.853, P = 0.039 for multivariate analysis, Tables 3 and 4). This is evidence that the Cdk1 N/C expression ratio could be an independent prognostic marker for colorectal cancer.

**Table 3:** **Univariate analysis of the influence of various parameters on overall survival in colon cancer patients**

Parameter	Category	Overall survival			
		5-year survival (%)	HR	95% CI	p value
Age, y	≥65/<65	43.9/45.7	1.261	0.899-1.769	0.179
Gender	Male/Female	44.8/44.4	1.134	0.811-1.586	0.463
Stage	II + III + IV/I	40.0/73.9	1.783	1.071-2.966	0.026
Cdk1 N/C ratio	≥1.5/<1.5	26.3/46.9	1.733	1.051-2.856	0.031

**Table 4:** **Multivariate analysis of the influence of various parameters on overall survival in colon cancer patients**

Parameter	Category	Overall survival			
		5-year survival (%)	HR	95% CI	p value
Age, y	≥65/<65	43.9/45.7	1.306	0.925-1.844	0.130
Gender	Male/Female	44.8/44.4	1.089	0.767-1.545	0.634
Stage	II + III + IV/I	40.0/73.9	1.712	1.027-2.853	0.039
Cdk1 N/C ratio	≥1.5/<1.5	26.3/46.9	1.712	1.027-2.853	0.039

Adjusted for age, gender and stage.

## Discussion

Cdk1 is a catalytic subunit that promotes the M-phase process and is essential for G1/S and G2/M phase transitions during cell proliferation [6, 20]. In addition, cyclin B-Cdk1 has been implicated in cell survival during the mitotic checkpoint (also known as the spindle assembly checkpoint) [21]. Cancer cells with high Cdk1 expression appear to have greater cell proliferation capability, so that patients with this type of tumor might have poor outcomes [5]. Thus, the use of small-molecule Cdk1 inhibitors could improve arrest at the G2-M boundary and prevent the entry of cancer cells into mitosis, thereby resulting in enhancement of apoptosis and tumor regression [21, 22]. These results suggest that Cdk1 might be more than just a therapeutic target but might serve as a predictor of the prognosis of cancer patients.

Cdk1 has been identified as a clinically useful prognostic marker in non-small cell lung cancer, colon cancer, and breast cancer [9–15]. High Cdk1 expression was associated with poor prognosis for cancer relapse, especially in node-negative breast cancer patients [9]. Non-small cell lung cancer patients with high Cdk1 expression levels also had poorer overall and recurrence survival than those with lower Cdk1 expression [12]. Interestingly, Zhang and colleagues found that although Cdk1 was highly expressed in non-small cell lung cancer tissues, its loss from the cytoplasm predicted poor survival and conferred resistance to chemotherapy both *in vivo* and *in vitro* [13]. This result suggested that Cdk1 might have different biological roles depending on its expression in the nucleus and cytoplasm and this might give rise to different clinical outcomes.

The prognostic role of Cdk1 in colon cancer is still controversial. For example, Zeestraten and colleagues identified that Cdk1 but not Cdk2 can predict distant-metastasis-free survival and cause-specific survival in stage II colon cancer [14]. Patients with high Cdk1 specific activity had significantly shorter distant-metastasis-free intervals compared with those with low Cdk1 specific activity [14]. Although the Cdk1 expression had no association with ki-67 mitotic index, the prognostic results were further supported by the enhanced expression of Cdk1 in microsatellite-stable tumors [14]. These findings were further supported by poor prognosis of colon cancer patients with microsatellite-stable tumors [23]. However, Meyer and colleagues reported that the absence of Cdk1 expression by immunohistochemistry staining of tissue arrays was associated with significantly poor cancer-related 5-year survival in stage UICC II colon carcinoma [15]. This study included patients with stage UICC II, III, and IV, and the prognostic role of Cdk1 in cancer-related 5-year survival risk was only observed in stage II but not in the other stages or when all stages were combined [15]. These conflicting results prompted us to investigate the prognostic role of Cdk1 in our patient population.

Considering the report that loss of cytoplasmic Cdk1 expression predicts poor clinical outcome in non-small cell lung cancer patients, we scored Cdk1 expression separately in the nucleus and cytoplasm [13]. In our cases, all specimens had Cdk1 expression in cytoplasm and only 2 cases had no Cdk1 expression in nucleus. No Cdk1 expression was found in adjacent normal tissue. Cox regression analysis of our data indicated no significant association of tumor Cdk1 expression in the nucleus and cytoplasm with clinical outcome (Additional file 3: Table S1). Kaplan–Meier analysis gave a similar result (Additional file 2: Figure S2).

The nuclear translocation of cyclin B/CDC2 complexes, which is required for the initiation of mitosis, is inhibited by 14-3-3σ. The 14-3-3 σ gene undergoes frequent epigenetic silencing in several types of cancer due to aberrant CpG methylation of specific promoters. Therefore we combined cytoplasmic and nuclear expression of Cdk1 as the N/C ratio for further analysis [19]. Interestingly, patients with high N/C ratios had lower 5-year survival rates than those with low N/C ratios (Table 3). Multivariate analysis confirmed the independent prognostic role of Cdk1 when grouped by N/C ratio (Table 4). This might reflect the poor prognosis of non-small cell lung cancer patients with tumors that lost cytoplasm Cdk1 expression [13].

The present study reveals a novel strategy for the use of the Cdk1 N/C ratio rather than the Cdk1 expression in the entire cancer cell for predicting prognosis of colon cancer patients. Our results suggested that Cdk1 might have a different prognostic role depending on the cancer type and the location of Cdk1 expression.

## Conclusions

In this study, we found no prognostic role of Cdk1 expression in the cytoplasm and nucleus, respectively. However, Cdk1 N/C expression ratio determined by IHC staining could be an independent prognostic marker for colorectal cancer.

## Electronic supplementary material

Additional file 1: Figure S1: Positive and negative control of Cdk1 IHC staining. **(A)** Normal colon tissue was used as the positive control and showed weak Cdk1 immunostain. Also seen was some Cdk1 positive lymphocytes infiltration in the colon tissues. **(B)** PBS was used instead of primary antibodies as a negative control. The same normal colon tissue core showed no Cdk1 immunoreactivity including colon glands and lymphocytes. (Magnification: 200×). (TIFF 5 MB)

Additional file 2: Figure S2: Kaplan-Meier actuarial analysis of overall survival according to Cdk1 expression in **(A)** cytoplasm and **(B)** nucleus in colorectal cancer patients. (TIFF 443 KB)

Additional file 3: Table S1: Univariate and multivariate analysis of cytoplasm and nucleus Cdk1 expression on overall survival in colorectal cancer patients. (DOCX 15 KB)
