# Supplementary figures and images for: High nuclear/cytoplasmic ratio of Cdk1 expression predicts poor prognosis in colorectal cancer patients
Source: BMC Cancer. 2014 Dec 15;14:951. doi: 10.1186/1471-2407-14-951 (PMC4302138; doi:10.1186/1471-2407-14-951)

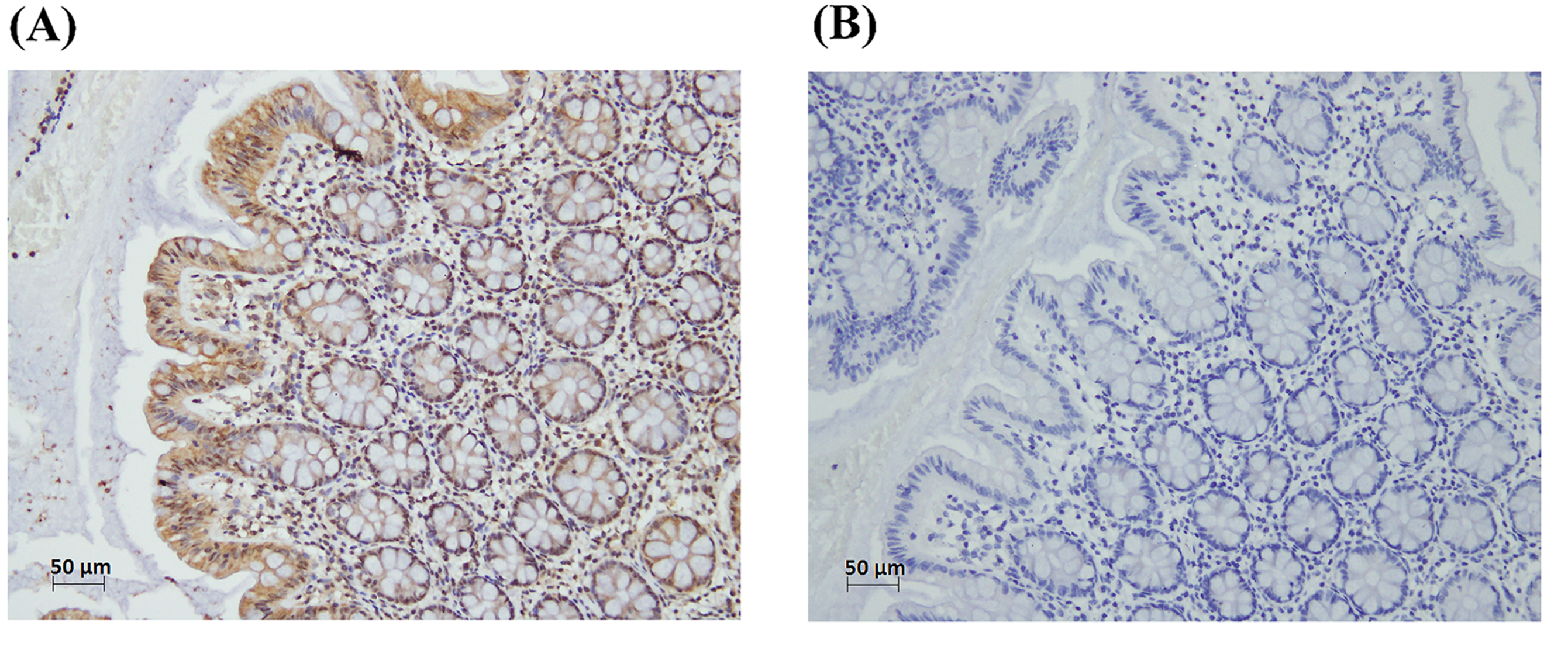

Supplement: Supplementary file 1 — Additional file 1: Figure S1: Positive and negative control of Cdk1 IHC staining. (A) Normal colon tissue was used as the positive control and showed weak Cdk1 immunostain. Also seen was some Cdk1 positive lymphocytes infiltration in the colon tissues. (B) PBS was used instead of primary antibodies as a negative control. The same normal colon tissue core showed no Cdk1 immunoreactivity including colon glands and lymphocytes. (Magnification: 200×). (TIFF 5 MB) [file 12885_2014_5103_MOESM1_ESM.tiff]

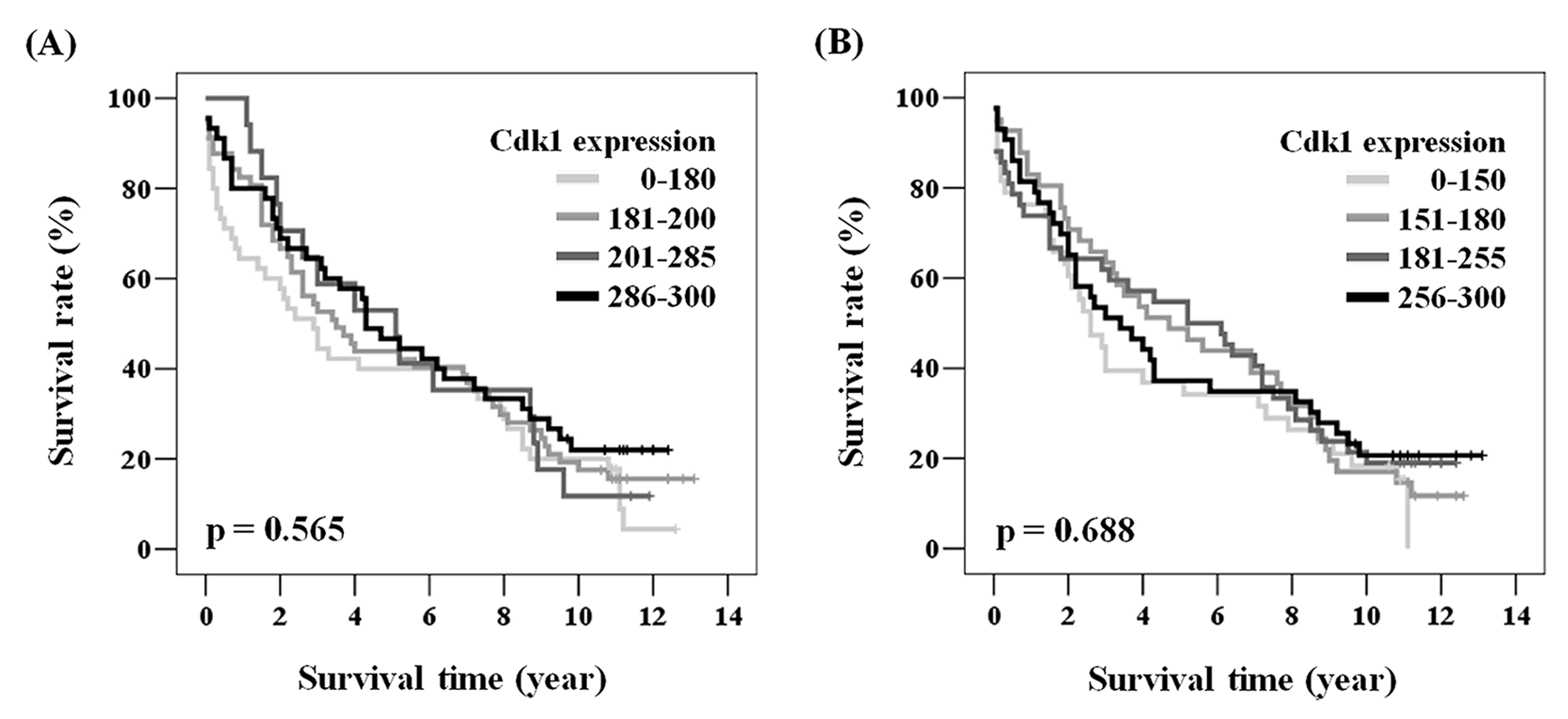

Supplement: Supplementary file 2 — Additional file 2: Figure S2: Kaplan-Meier actuarial analysis of overall survival according to Cdk1 expression in (A) cytoplasm and (B) nucleus in colorectal cancer patients. (TIFF 443 KB) [file 12885_2014_5103_MOESM2_ESM.tiff]
