# Supplementary material for: High nuclear/cytoplasmic ratio of Cdk1 expression predicts poor prognosis in colorectal cancer patients
Source: BMC Cancer. 2014 Dec 15;14:951. doi: 10.1186/1471-2407-14-951 (PMC4302138; doi:10.1186/1471-2407-14-951)
Supplement: Supplementary file 3 — Additional file 3: Table S1: Univariate and multivariate analysis of cytoplasm and nucleus Cdk1 expression on overall survival in colorectal cancer patients. (DOCX 15 KB) [file 12885_2014_5103_MOESM3_ESM.docx]

Supplementary Table 1. Univariate and multivariate analysis of cytoplasm and nucleus Cdk1 expression on overall survival in colorectal cancer patients.

|  | Overall survival | | | | | | | |
| --- | --- | --- | --- | --- | --- | --- | --- | --- |
|  |  | Univariate analysis | | |  | Multivariate analysis* | | |
| Expression value | 5-year survival (%) | HR | 95% CI | p value |  | HR | 95% CI | p value |
| Cytoplasm Cdk1 |  |  |  |  |  |  |  |  |
| 0-180 | 40.0 | 1.000 | Referent |  |  | 1.000 | Referent |  |
| 181-200 | 43.9 | 0.847 | 0.557-1.290 | 0.440 |  | 0.836 | 0.548-1.276 | 0.407 |
| 201-285 | 52.9 | 0.819 | 0.452-1.484 | 0.520 |  | 0.799 | 0.440-1.452 | 0.462 |
| 286-300 | 46.7 | 0.723 | 0.459-1.139 | 0.162 |  | 0.705 | 0.444-1.122 | 0.140 |
| Nucleus Cdk1 |  |  |  |  |  |  |  |  |
| 0-150 | 36.8 | 1.000 | Referent |  |  | 1.000 | Referent |  |
| 151-180 | 48.8 | 0.815 | 0.510-1.304 | 0.394 |  | 0.792 | 0.492-1.275 | 0.337 |
| 181-255 | 54.8 | 0.775 | 0.481-1.248 | 0.294 |  | 0.729 | 0.452-1.178 | 0.197 |
| 256-300 | 37.2 | 0.785 | 0.488-1.264 | 0.320 |  | 0.752 | 0.466-1.215 | 0.244 |

*Adjusted for age, gender and stage.
